# Spectroelectrochemical studies on the effect of cations in the alkaline glycerol oxidation reaction over carbon nanotube-supported Pd nanoparticles

**DOI:** 10.3762/bjoc.14.120

**Published:** 2018-06-12

**Authors:** Dennis Hiltrop, Steffen Cychy, Karina Elumeeva, Wolfgang Schuhmann, Martin Muhler

**Affiliations:** 1 Laboratory of Industrial Chemistry, Ruhr-Universität Bochum, Universitätsstr. 150, 44780 Bochum, Germany; 2 Analytical Chemistry - Center for Electrochemical Sciences (CES), Ruhr-Universität Bochum, Universitätsstr. 150, 44780 Bochum, Germany

**Keywords:** cation effect, electrocatalysis, glycerol oxidation, in situ electrochemistry/IR spectroscopy

## Abstract

The effects of the alkali cations Na^+^ and K^+^ were investigated in the alkaline electrochemical oxidation of glycerol over Pd nanoparticles (NPs) deposited on functionalized carbon nanotubes (CNTs). The electrocatalytic activity was assessed by cyclic voltammetry revealing a lower overpotential of glycerol oxidation for nitrogen-functionalized Pd/NCNTs compared with oxygen-functionalized Pd/OCNTs. Whereas significantly lower current densities were observed for Pd/OCNT in NaOH than in KOH in agreement with stronger non-covalent interactions on the Pd surface, Pd/NCNT achieved an approximately three-times higher current density in NaOH than in KOH. In situ electrochemistry/IR spectroscopy was applied to unravel the product distribution as a function of the applied potential in NaOH and KOH. The IR spectra exhibited strongly changing band patterns upon varying the potential between 0.77 and 1.17 V vs RHE: at low potentials oxidized C_3_ species such as mesoxalate and tartronate were formed predominantly, and with increasing potentials C_2_ and C_1_ species originating from C–C bond cleavage were identified. The tendency to produce carbonate was found to be less pronounced in KOH. The less favored formation of highly oxidized C_3_ species and of carbonate is deduced to be the origin of the lower current densities in the cyclic voltammograms (CVs) for Pd/NCNT in KOH. The enhanced current densities in NaOH are rationalized by the presence of Na^+^ ions bound to the basic nitrogen species in the NCNT support. Adsorbed Na^+^ ions can form complexes with the organic molecules, presumably enhanced by the chelate effect. In this way, the organic molecules are assumed to be bound more tightly to the NCNT support in close proximity to the Pd NPs facilitating their oxidation.

## Introduction

The conversion of biomass to biofuels is a promising process for carbon-neutral energy conversion [1]. First-generation biofuels such as bioethanol or biodiesel are produced from starch, sugar or oils and fats [2–3]. The transesterification of oils and fats produces glycerol as a byproduct, which requires further processing to reduce the costs of biofuels [4]. The comparably high energy density of glycerol of 5965 Wh L^−1^ assuming its total oxidation to CO_2_ suggests its utilization in fuel cells (FCs) converting chemical into electrical energy [5]. In alkaline FCs (AFCs) and proton exchange membrane FCs (PEMFCs), H_2_ and O_2_ are converted to H_2_O, heat and electricity. Since the development of anion exchange membranes the research interest in AFCs has increased again, because AFCs have several advantages over PEMFCs such as faster kinetics of the oxygen reduction reaction at the cathode, greater longevity and the possible adaption of less precious metals due to the alkaline electrolyte [6–7]. In direct alkaline alcohol FCs (DAAFCs) electricity is generated by oxidizing small organic molecules. A direct alkaline methanol FC was already tested in 1965 oxidizing methanol over a Pt catalyst in highly alkaline medium [8]. Alcohols such as ethanol and glycerol are valuable and non-toxic substrates for DAAFCs, too. However, the higher chemical complexity of the alcohols complicates the total oxidation reaction, because numerous intermediates occur that need to be oxidized efficiently without poisoning the catalyst.

Another important utilization of glycerol is the electrosynthesis of fine chemicals. It is a highly suitable precursor for valuable chemicals such as DL-glyceric acid, mesoxalic acid and 1,3-dihydroxyacetone (DHA). These products are conventionally obtained by oxidation using rather unselective oxidants. Especially DHA is of great interest due to its use in the cosmetic industry, as a precursor for further value-added fine chemicals [9] and as a monomer for biopolymers like polyesters and polyurethanes [4]. The route to higher functionalized products starting from glycerol via electrosynthesis offers several advantages compared with thermal gas-phase oxidation processes. In the aqueous environment low temperatures and pressures are applied allowing a higher control of selectivity. Moreover, additional parameters such as pH value, the nature of the ions and the concentration of glycerol in the liquid phase can be tuned in liquid-phase processes [4].

Monitoring of electrocatalytic reactions is challenging as all products are dissolved in the electrolyte. High-performance liquid or ion chromatography and differential electrochemical mass spectrometry provide information about the nature of dissolved and chemically stable products [10–11]. A rather direct view on the reaction is provided by infrared (IR) spectroscopy. Specifically designed experimental cells focus the IR beam on the working electrode surface through a thin electrolyte layer probing vibrations of adsorbed intermediates and products [12]. Highly active catalysts converting glycerol to carbonate consume 20 hydroxide ions per glycerol molecule in the thin electrolyte layer, which leads to a substantial local pH drop and cannot be fully compensated by OH^−^ diffusion from the bulk into the thin layer. This pH drop further complicates the identification of products, because the product selectivity may change as a function of pH as demonstrated by Wang et al. [13] on a Pd disk electrode for ethylene glycol electrooxidation. For glycerol, a similar study was conducted by Ferreira et al. [14] on PdRh electrodeposits showing that the glycerol oxidation route follows a pathway involving the consumption rather than production of H_2_O once the initially present OH^−^ ions are consumed. Finally, the structural similarity of the possible oxidation products makes the assignment of occurring IR bands to certain species challenging. Glycerol oxidation was studied over different catalytically active metals, such as Au [15], AuAg/C [16], Pt [17–18], Au/C [19–20], Pt/C [20–21], Pd/C [20], PdAu/C [22], PdNi/C, PdAg/C [23] and PtBi/C [21] in alkaline medium using IR spectroscopy for product identification.

Strmcnik et al. [24] investigated the effect of different alkali ions using LiOH, NaOH, KOH and CsOH on the observed currents in the hydrogen oxidation, oxygen reduction and methanol oxidation reactions over Pt single crystal, polycrystalline Pt and Pt/C electrodes. They proposed two different adsorbate structures of hydrated alkali metal cations on the electrode, either via a hydrogen bonding between hydrating water and two OH_ad_ groups (1) or direct ion–dipole interactions between two OH_ad_ and the cation (2). The formation of the structures (1) or (2) was correlated to the hydration energies (Δ*H*_M_) of the cations. Highly charged species with a high Δ*H*_M_ like Li^+^ were considered more likely to form structure (2), because the cation–OH_ads_ interaction is stronger than for cations with a low Δ*H*_M_ like Cs^+^. A high surface coverage with such clusters was assumed to inhibit the adsorption and movement of reactants on the electrode surface accounting for the activity trends Li^+^ < Na^+^ < K^+^ < Cs^+^ in the investigated reactions [24].

This study gave rise to further investigations on the impact of ions on electrochemical reactions. Studies of the cation effect on Au electrodes are scarce [25–26], as the majority of reports employ Pt electrodes. In acidic medium the influence of Cs^+^ and Na^+^ on the specific adsorption of OH, O and H on polycrystalline Pt was investigated [27] as well as the cation influence on the water electrolysis on Pt(111) [28] and Ir(111)/oxide electrodes [29]. Different Pt single crystal surfaces [30] and other electrochemical reactions such as CO [30], formate [31] and ethylene glycol oxidation [32–33] were studied as well. Especially for these more complex oxidation reactions of small organic molecules, studies of the cation impact on the formed products are rather scarce. Sitta et al. [32] revealed an analogous trend as Strmcnik et al. [24], i.e., increasing current densities for ethylene glycol electrooxidation over Pt in the order Li^+^ < Na^+^ < K^+^. They used IR spectroscopy to determine the nature of the formed products and identified carbonate and oxalate as the main products in addition to traces of glycolate and CO. By comparing the ratio of the integrated band intensities for oxalate and carbonate they concluded that C–C bond scission is influenced by the cation in the electrolyte by blocking surface sites that are required for the carbon bond cleavage.

To the best of our knowledge, there are no studies focusing on the cation effect on the glycerol electrooxidation reaction using Pd-based materials. With respect to FC applications and the electrosynthesis of fine chemicals it is important to extend the knowledge about this effect. In this study we used Pd nanoparticles (NPs) deposited on functionalized carbon nanotubes (Pd/xCNT, x = N or O) for catalyzing the glycerol oxidation reaction (GOR). Pd/NCNT has been investigated recently in the ethanol electrooxidation reaction in alkaline medium reaching high specific current densities of about 500 A g^−1^_Pd_ [34]. The Pd/OCNTs of this study differ from the Pd/OCNTs used here by an additional He treatment of the oxygen-functionalized CNTs to improve the comparability of the catalysts due to the same thermal treatment. Cyclic voltammetry was used to determine the glycerol oxidation activity of the catalysts, and electrochemistry coupled with attenuated total reflection IR (ATR–IR) spectroscopy was performed to determine the product distribution in the thin electrolyte layer between the working electrode and the ATR crystal. The band assignment was supported by reference measurements under identical conditions.

## Results and Discussion

### Electrochemical glycerol oxidation

**Figure 1:**
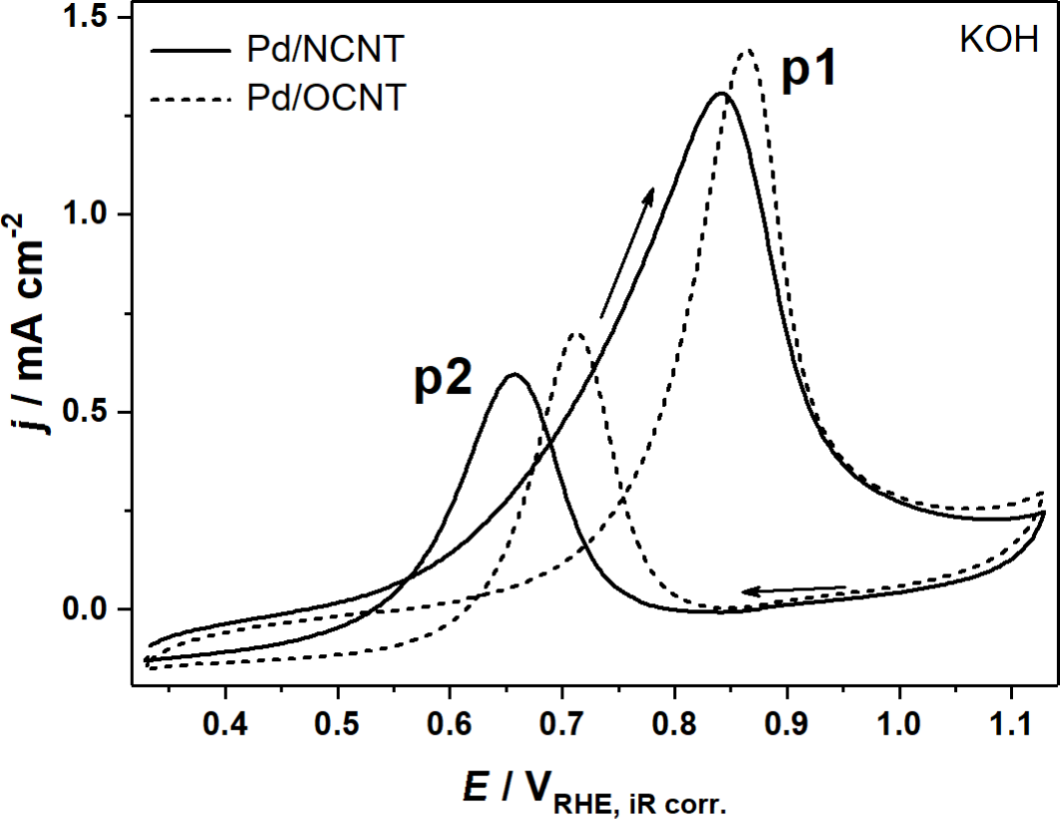
CVs of the electrooxidation of 1 M glycerol over Pd/NCNT and Pd/OCNT in 1 M KOH at 1000 rpm at a scan rate 0.01 V s^−1^.

Figure 1 shows cyclic voltammograms (CVs) of the two catalysts obtained in 1 M KOH containing 1 M glycerol. In the forward scan the peak labeled p1 occurs due to the oxidation of glycerol on the Pd^0^ NPs, which is inhibited at *E* > 0.9 V vs RHE by PdO formation. In the reverse scan, the oxide layer is reduced back to Pd^0^ at around 0.8 V vs RHE. The oxidative current densities at *E* < 0.8 V vs RHE are then caused by the oxidation of freshly adsorbing glycerol as well as oxidation of still adsorbed species from the forward scan (p2). Quantitative information extracted from the CVs in NaOH and KOH is summarized in Table 1.

**Table 1:** Peak potentials *E*_p1_ and *E*_p2_, peak current densities *j*_p1_ and *j*_p2_, and peak charges *Q*_p1_ and *Q*_p2_ extracted from the CVs shown in Figure 1 and Figure 2.

	Pd/OCNT		Pd/NCNT	
	KOH	NaOH	KOH	NaOH

*E*_p1_, *E*_p2_ [V] vs RHE	0.86, 0.71	0.88, 0.71	0.84, 0.66	0.84, 0.66
*j*_p1_, *j*_p2_ [mA cm^−2^]	1.42, 0.70	0.55, 0.19	1.31, 0.59	3.08, 1.74
*Q*_p1_, *Q*_p2_ [C]	17.3, 7.09	6.80, 2.16	26.0, 7.58	62.5, 25.6

The CVs illustrate the differences between the two catalysts. For Pd/NCNT peak p1 is much broader and the peak potentials (*E*_p1_ and *E*_p2_) are found at lower anodic potentials (−29 and −52 mV vs RHE on average) compared with Pd/OCNT. Although the peak current densities are almost equal, the catalytic activity towards glycerol oxidation is higher for Pd/NCNT, as the reaction starts at lower anodic overpotentials. This activity relation is in good agreement with studies applying these catalysts in ethanol electrooxidation and olefin hydrogenation [34–35].

**Figure 2:**
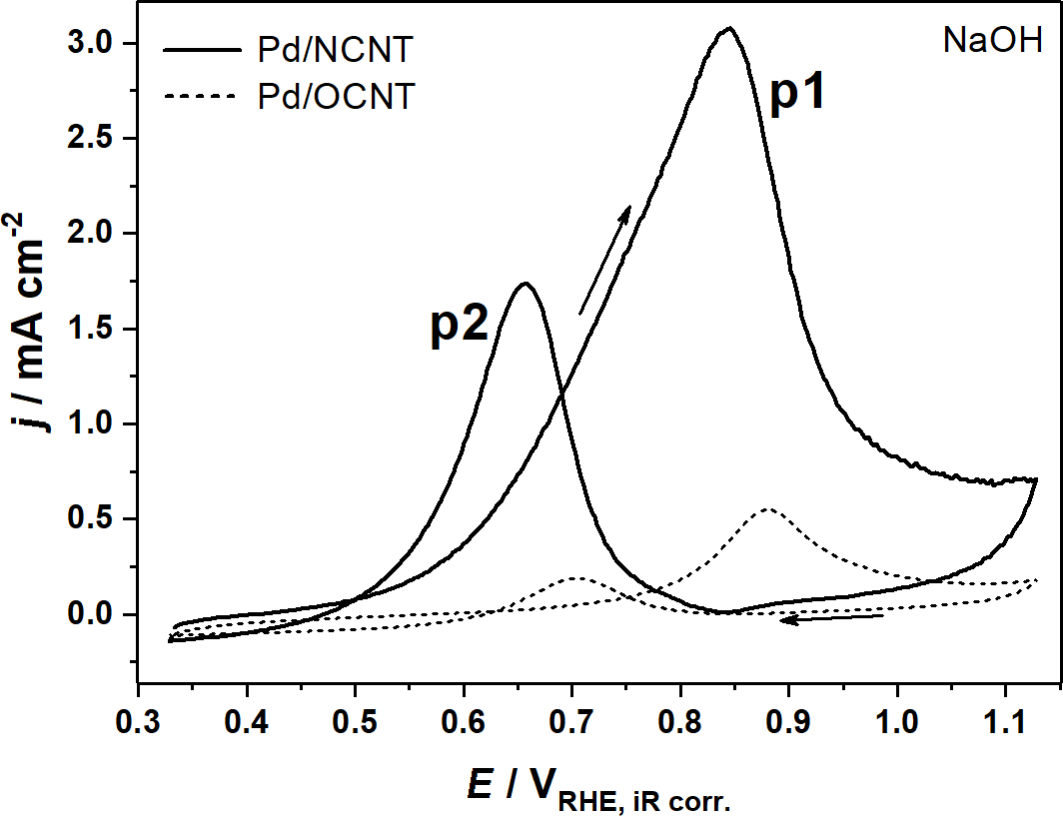
CVs of the electrooxidation of 1 M glycerol over Pd/NCNT-NH_3_ and Pd/OCNT-He in 1 M NaOH at 1000 rpm at a scan rate 0.01 V s^−1^.

Analogous CV experiments were also carried out in NaOH and the obtained CVs are shown in Figure 2. The current densities recorded for Pd/OCNT are significantly lower in NaOH compared with KOH in agreement with the results reported by Strmcnik et al. [24]. Surprisingly, the current densities for Pd/NCNT are enhanced by a factor of approximately 2.4 without significant changes in peak potentials and shapes. Obviously, the peak potentials and shapes depend on the structural properties of the catalyst, whereas the changes in *j*_p1_ and *j*_p2_ are caused by the nature of the cation in the electrolyte.

The higher catalytic activity of the metallic Pd NPs supported on NCNTs in heterogeneous gas-phase catalysis has been explained by an electronic effect due to the electron-donating effect of the embedded nitrogen species [35]. Pd^0^ is also considered the active site in glycerol electrooxidation, and the observed lower overpotential may originate from the same interaction with the nitrogen-doped support. Furthermore, the thermal treatment of the oxygen-functionalized CNTs in He reduces the number of acidic groups on the surface leading to a higher degree of hydrophobicity of the OCNT support lowering the coverage of adsorbed glycerol. A combination of these properties may explain the lower activity of Pd/OCNT, but it does not provide a straightforward explanation for the observed higher current densities for Pd/NCNT in NaOH compared with KOH considering the non-covalent interactions proposed by Strmcnik et al. [24].

### In situ FTIR spectroscopy

**Figure 3:**
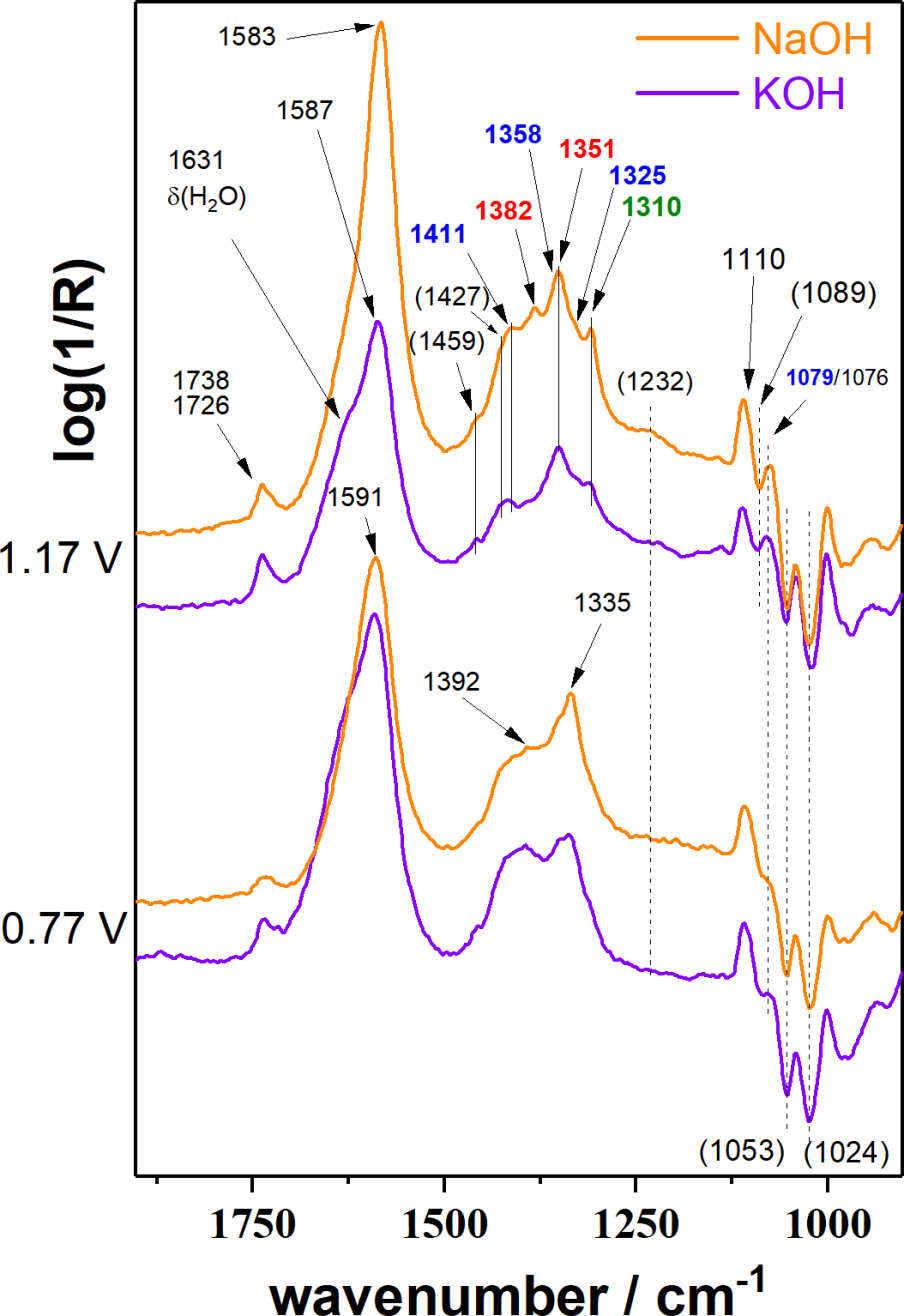
Comparison of IR spectra recorded at 0.77 and 1.17 V vs RHE (further potentials are shown in Supporting Information File 1, Figure S2) for Pd/NCNT after electrooxidation of 1 M glycerol for 14.7 min in 0.1 M of NaOH or KOH, respectively (*d*_TL_ = 28 µm).

The reaction products in NaOH and KOH solutions formed over Pd/NCNT were identified by IR spectroscopy (Figure 3, Table 2) using a cell that allows to adjust the distance between the electrode surface and the IR window (*d*_TL_) [36]. The distance *d*_TL_ was adjusted to 28 µm to minimize the impact of local pH changes ensuring bulk-like reaction conditions in the thin layer (Supporting Information File 1, Figure S1). At 0.77 V vs RHE the spectra in NaOH and KOH appear to be quite similar. The most pronounced difference is the intense band at 1335 cm^−1^ in NaOH. A shoulder at 1351 cm^−1^ found in both electrolytes is assigned to the ν_s_(COO^−^) vibration of formate [37]. We assign the band at 1335 cm^−1^ to a highly oxidized C_3_ species like tartronate and mesoxalate [15], although in literature this signal is also often attributed to 1,3-dihydroxyacetone (DHA) [20–21,38–39]. Our reference experiments with DHA (Supporting Information File 1, Figure S3) exclude this assignment, because no band was found at 1335 cm^−1^ [15–16]. Further bands caused by DHA would occur at 1738 (ν(C=O)), 1056 and 1003 cm^−1^, of which only the first can be identified. The intensity is generally weak and found to decrease with higher potential suggesting that it is either less formed or more easily further oxidized.

**Table 2:** Wavenumbers of identified species found in the IR spectra. Wavenumbers in italics originate from homemade reference experiments, other species were identified using refs. [15–16] if not indicated differently.

identified species	molecular structure	wavenumber^a^ [cm^−1^]

methanol	H_3_COH	*1110*
formate	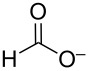	*1382*, *1351*
carbonate	CO_3_^2−^	*1390*
glycolate, glycerate	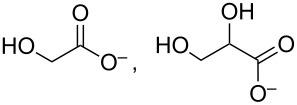	*1411* (sh), *1358* (sh), *1325* (sh)
oxalate	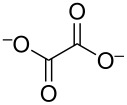	*1310*
glyoxal, glyoxylate	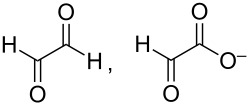	1076 [13]
glycerol	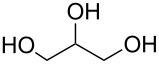	(1112**,** 1044)
tartronate, mesoxalate	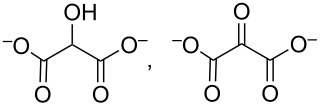	1725 (sh), 1335, 1110
DHA	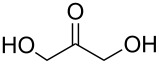	*1738* (*1056*, *1003*)

^a^“sh” is an abbreviation for “shoulder”; wavenumbers in brackets indicate expected bands that were not identified in the IR spectra.

The assignment to tartronate or mesoxalate is additionally supported by the presence of bands at 1110 and 1725 cm^−1^ (shoulder of the band at 1738 cm^−1^). Hydroxypyruvate (1383, 1215 cm^−1^ [15]) is tentatively excluded as no sharp band intensity is found. The intense slightly asymmetric band around 1590 cm^−1^ cannot be assigned to a single species, because most of the possible products contain one or more carboxylic groups. In addition, there is the overlap with the H_2_O bending mode (δ(H_2_O)) at ≈1631 cm^−1^. The negative signals at 1053 and 1024 cm^−1^ indicate the consumption of a compound but do not correspond to the ν(C–O) bands of glycerol (1105, 1060 cm^−1^) rendering their assignment difficult.

Upon increasing the applied potential to 1.17 V vs RHE, the IR spectra in both NaOH and KOH change. The band at 1335 cm^−1^ is hardly visible anymore, but additional bands are identified and assigned to δ(HCO) of formate (1382 cm^−1^, only in NaOH) [37] and oxalate (1310 cm^−1^) [15–16]. Glycerate and glycolate are presumably also present. Both contain one fully oxidized terminal C atom and by cleaving the C–C bond in glycerate, glycolate can be obtained. Thus, the easiest differentiation would be by a ν(C–O) band at around 1125 cm^−1^ [15]. This band is not found, but glycolate bands (wavenumbers in blue in Figure 3) [15] are present as shoulders indicating that glycerate is either oxidized further too fast to be observed or glycolate is generated from a different species like tartronate after C–C cleavage. A weak band at 1076 cm^−1^ could indicate glyoxal or glyoxylate that are only found at higher potentials as intermediate species towards oxalate [13].

The product assignment clearly indicates that increasing potentials favor C–C bond cleavage. Bond scission is likely to proceed via highly oxidized C_3_ species that are prone to undergo C–C scission more easily as the carbon atoms become positively charged with increasing number of oxygen atoms in the molecule. An additional indicator is the band at 1110 cm^−1^ already assigned to mesoxalate or tatronate. It is observed that its IR intensity is slightly higher at 1.17 V vs RHE. Because C_3_ species are less favored at higher potentials, this band has to be at least partly caused by another species. A reasonable assignment is the ν(C–O) vibration of a methoxy group. For instance, the glycerate-to-glycolate conversion generates a methoxy species for each converted molecule. For the NaOH electrolyte this band is increasing with more anodic potential supporting this hypothesis.

The generation of C_1_ species further suggests the production of carbonate (1390 cm^−1^ [15]). Its contribution seems to be rather weak at 0.77 V vs RHE, whereas the spectra recorded at 1.17 V vs RHE show a contribution. Especially the spectrum in KOH shows a strong lack of intensity in this region in comparison to the spectrum in NaOH. The conclusions drawn from the IR spectra help to explain the CV results. The last oxidation step of glycerol to carbonate via three formate species involves almost 43% of the transferable number of electrons per mole of glycerol. Thus, the lack of band intensity in the carbonate region in KOH in combination with the generally lower IR band intensities indicate an overall lower degree of glycerol conversion and extent of oxidation producing carbonate as final product.

The surface coverage of solvated Na^+^ clusters on the Pd NPs is supposed to be higher than for the corresponding K^+^ clusters and thus should hinder the adsorption/oxidation of glycerol leading to lower currents also for Pd/NCNT [24]. It has to be pointed out that a non-linearity of this cation effect has already been found for Au electrodes in the case of ethanol and methanol oxidation [26] and H_2_O electrolysis in alkaline media [28]. In our case using CNT-supported Pd NPs, the observed cation-related activity trend suggests another reason due to the differently functionalized CNT surfaces. Gosselink et al. [40] reported on the beneficial role of increased surface polarity of carbon nanofibers introduced by oxygen groups in the deoxygenation of stearic acid over Pd NPs deposited in addition on this support. They identified a favorable mode of adsorption of the stearic acid via the carboxylic group for high support polarity instead of a flat adsorption mode for low support polarity [40]. The upright adsorption mode facilitates the deoxygenation due to the close contact with the Pd NPs pointing to the importance of metal-support interactions [40].

A similar adsorption-induced effect may be present in glycerol oxidation over Pd/NCNT in NaOH due to the more favorable interaction of Na^+^ with the basic nitrogen species in the CNT surface acting as ligands. Also the oxygen-containing functional groups in glycerol and the various intermediates can act as ligands resulting in a high affinity to the complexing Na^+^ ions on the NCNT support. Thus, oxidation over the Pd NPs is facilitated, because the organic species are kept close to the catalytically active Pd NPs. As a result, highly oxidized products are formed leading to higher current densities. The chelate effect is assumed to strengthen the binding of the oxygen-functionalized molecules acting as polydentate ligands. We plan to extend our experimental series to LiOH and CsOH and to apply Pd NPs deposited on non-functionalized CNTs and Pd film electrodes to provide additional support for the postulated beneficial role of Na^+^ surface complexes on NCNTs.

## Conclusion

The effects of the alkali cations Na^+^ and K^+^ on the electrochemical oxidation of glycerol were investigated over Pd nanoparticles supported on carbon nanotubes. The CNTs used as support for the Pd NPs were either oxygen- or nitrogen-functionalized. Cyclic voltammetry showed that the achieved current densities were higher in KOH than in NaOH solution for Pd/OCNT in agreement with the theory of weaker non-covalent interactions on the Pd surface in KOH. Pd/NCNT was electrocatalytically more active as shown by the lower anodic onset potential achieving slightly higher current densities in KOH compared with Pd/OCNT. In contrast, significantly higher current densities in NaOH were observed for Pd/NCNT. The product distribution determined by ATR–IR spectroscopy at constant potentials revealed significant differences considering the relative production of species. The IR spectra recorded in NaOH indicate the presence of more strongly oxidized products including C–C bond scission. More specific, in KOH less IR intensity in the carbonate band region was found in agreement with the lower current densities observed during the CV experiments. It is claimed that the Na^+^ cations interact with the nitrogen-functionalized CNTs more strongly forming complexes with the organic molecules, presumably enhanced by the chelate effect. In this way, the organic molecules are assumed to be bound more tightly to the NCNT support in close proximity to the catalytically active Pd NPs facilitating their oxidation.

## Experimental

### Catalysts

The preparation of the two catalysts Pd/NCNT and Pd/OCNT has been published elsewhere [34–35,41]. In brief, the oxygen- and subsequent nitrogen-functionalization of the purified and washed CNTs (Baytubes C 150 P, washed in 1.5 M HNO_3_) was performed by HNO_3_ vapor treatment at 473 K for 48 h. Then, the obtained O-functionalized CNTs were exposed to 10 vol % of NH_3_/He at 673 K for 6 h to obtain NCNTs. The O-functionalized CNTs were additionally heated in He at 673 K for 6 h (OCNTs). The Pd NPs were deposited from a colloidal method, dried at 333 K for 12 h, reduced in 10 vol % H_2_/He (100 mL min^−1^) for 2 h and cooled to room temperature in He. The catalysts possess a weight load of <1 wt %.

### Electrochemistry

The electrochemical characterization of Pd/NCNT and Pd/OCNT was performed in a conventional three-electrode set-up using 0.11 cm^2^ glassy carbon electrodes as working electrode, a Ag/AgCl/3 M KCl as reference and a Pt mesh as counter electrode. The working electrodes were polished to a mirror finish using grinding papers and alumina suspensions of different grain sizes. Prior to each experiment, the glassy carbon surface was repolished using a 0.05 µm Al_2_O_3_ suspension and ultrasonicated in H_2_O before a new catalyst layer was deposited. The catalyst ink was prepared according to a final concentration of 5 mg/mL using 49:49:2 vol % H_2_O/ethanol/Nafion. An aliquot of the catalyst ink was drop-cast on the glassy carbon electrode achieving a loading of 210 µg cm^−2^ and dried in air at ambient conditions. The catalyst-covered electrodes were initially conditioned in a potential range from 0.33 to 1.13 V vs RHE in Ar-saturated 1 M of alkali base + 1 M of glycerol for 10 cycles at a scan rate of 0.1 V s^−1^ and an electrode rotation rate of 1000 rpm. The uncompensated electrolyte resistance was determined by electrochemical impedance spectroscopy performed at open circuit potential without electrode rotation before glycerol oxidation was monitored using a scan rate of 0.01 V s^−1^ for one cycle. The results were repeated to ensure reproducibility. The potential was applied by a Metrohm Autolab potentiostat and is reported versus the reversible hydrogen electrode scale.

### Spectroelectrochemistry

FTIR experiments were performed in a spectroelectrochemical cell described in detail in ref. [36]. In brief, it combines ATR–IR spectroscopy with electrochemical techniques in a thin electrolyte layer configuration, where the thickness of the thin layer is tunable in the micrometer range by an implemented micrometer screw. The Bruker Tensor 27 FTIR spectrometer was equipped with an A530/P reflection unit, a liquid nitrogen mercury cadmium telluride detector and was constantly purged with dried air. All IR experiments were performed in Ar-saturated 0.1 M alkali base containing 1 M glycerol using a Ge internal reflection element. The active electrode area was slightly increased to 0.5 cm^2^ to increase the IR band intensity of the generated species by covering a substantial part of the internal reflection element. The electrode preparation and catalyst deposition were performed in the same manner as for the other experiments yielding a comparable surface loading. If not otherwise mentioned, all IR spectra were recorded in the range from 4000 to 700 cm^−1^ with a resolution of 4 cm^−1^ and correspond to the average of 200 individual interferograms (≈90s) after automatic subtraction of the reference spectrum recorded with the same number of IR scans. The spectra are shown as log(1/reflection) with generated and consumed species pointing upwards and downwards, respectively, with respect to the reference spectrum. Spectra acquisition was controlled using the Opus 7.2 software. The constant sample potentials that were applied during the spectroelectrochemical experiments were modulated by a Metrohm Autolab PGSTAT204 potentiostat using the Nova 1.11.2 software. Reference spectra were recorded without adapting any electrochemical equipment. The compounds DHA, glyceric acid, carbonate, formate, oxalate, glycerol and methanol were diluted in 0.1 M KOH at a concentration of 0.05 M and several spectra were recorded to check for chemical stability in alkaline medium.

## Supporting Information

File 1 Additional spectra.
