# Supplementary material for: Spectroelectrochemical studies on the effect of cations in the alkaline glycerol oxidation reaction over carbon nanotube-supported Pd nanoparticles
Source: Beilstein J Org Chem. 2018 Jun 12;14:1428–35. doi: 10.3762/bjoc.14.120 (PMC6009201; doi:10.3762/bjoc.14.120)
Supplement: File 1 — Additional spectra. [file Beilstein_J_Org_Chem-14-1428-s001.pdf]

Supporting Information

for

Spectroelectrochemical studies on the effect of cations in the alkaline glycerol oxidation reaction over carbon nanotube-supported Pd nanoparticles

Dennis Hiltrop[1], Steffen Cychy[1], Karina Elumeeva[2], Wolfgang Schuhmann[2] and Martin Muhler*[1]

Address: [1]Laboratory of Industrial Chemistry, Ruhr-Universität Bochum, Universitätsstr. 150, 44780 Bochum, Germany and [2]Analytical Chemistry - Center for Electrochemical Sciences (CES), Ruhr-Universität Bochum, Universitätsstr. 150, 44780 Bochum, Germany

Email: Martin Muhler - muhler@techem.rub.de

* Corresponding author

Additional spectra

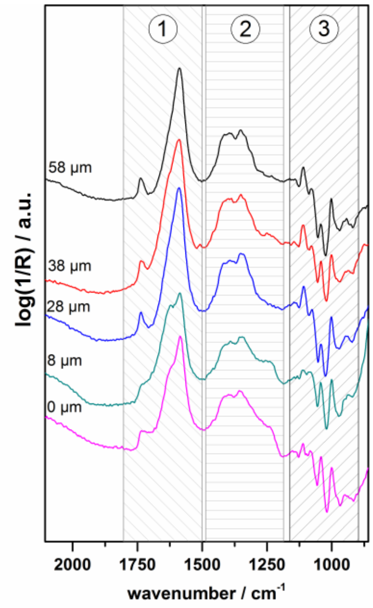

Figure S1: IR spectra recorded at decreasing d_{TL} after 13.2 min at 0.87 V vs RHE in 0.1 M KOH + 1 M glycerol. IR spectra were recorded during glycerol oxidation in KOH at different d_{TL} to highlight the change in the band patterns when the electrode is brought into too close proximity with the IR window. Three different regions are marked for better understanding. In region 3 mainly ν(C=O) of aldehydes and ketones (1730 cm^{-1}) and $\nu_{as}(COO^-)$ vibrations (1590 cm^{-1}) of carboxylic groups are present. Additionally, the $\delta(H_2O)$ vibration of water (1635 cm^{-1}) is overlapping with $\nu_{as}(COO^-)$. It is observed that the ν(C=O) vibration is merging with the intense band at 1600 cm^{-1} and $\delta(H_2O)$ is relatively increased the closer the electrode surface is brought to the IR window. Region 2 shows the appearance of a shoulder around 1250 cm^{-1}, a general broadening and decreasing IR band intensities of the complex pattern between 1500 and 1250 cm^{-1} upon decreasing d_{TL}. This region is more meaningful to determine the products formed in this reaction, as several different vibrations absorb IR radiation but with a higher separation as compared to region 1. The identification of the products in the main text is based on this region.

Region 3 shows the ν(C–O) vibration of the different OH-containing molecules formed by glycerol electrooxidation. Furthermore, it shows negative bands, which indicate vibrations of species that are being consumed. At 58 µm it is possible to distinguish increasing (1112 cm^{-1}) from decreasing bands (1055, 1024 cm^{-1}), which becomes impossible at distances < 10 µm. Furthermore, the initial sharp band at 1112 cm^{-1} is vanishing at close distances indicating that the product formation is depending on d_{TL}.

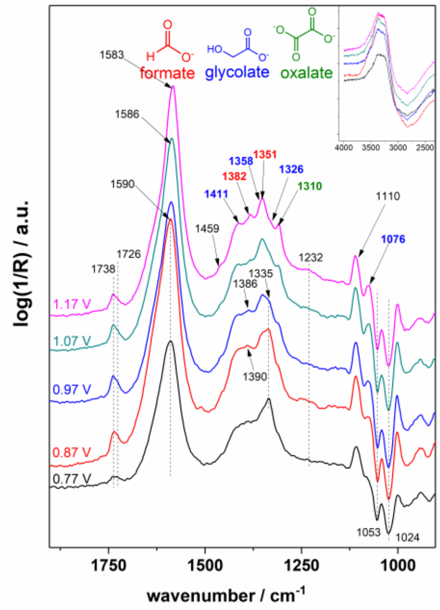

Figure S2: IR spectra recorded in 0.1 M NaOH + 1 M glycerol between 0.77 and 1.17 V vs RHE (ΔE_S = 0.1 V vs RHE) over Pd/NCNT after electrolyzing for 14.7 min (d_{TL} = 28 µm). The spectra at 0.77 V vs RHE and 1.17 V vs RHE are included in the main text in Figure 2. The additional spectra recorded at intermediate potential show the transition in terms of product distribution. It is clearly seen that the band at 1335 cm^{-1} is vanishing and replaced by the bands assigned to distinct species as mentioned in the main text.

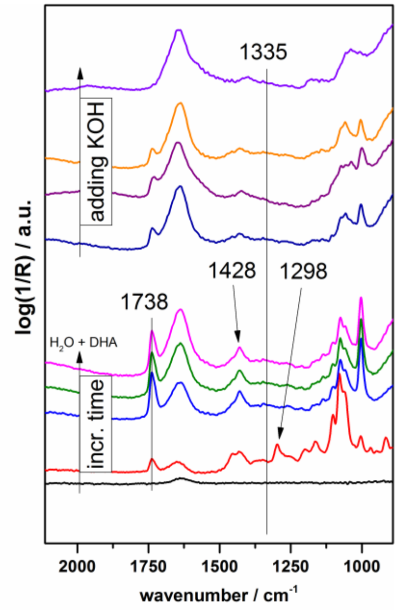

Figure S3: Reference spectrum of 0.05 M DHA in 0.1 M KOH starting at pH 7 and successively adding KOH. The reference spectrum shows the IR spectrum of DHA in alkaline solution. The different bands found in the red spectrum (directly after adding DHA to the pH 7 H_2O background, black line) are disappearing rapidly or change their intensity. Upon adding KOH to the H_2O/DHA mixture this change continues and the last spectrum shows only very broad bands. This experiment illustrates the high reactivity of DHA in solution. It is further supported by the observed coloring of the solution ranging from a clear solution to yellow/brownish depending on the initial base concentration.

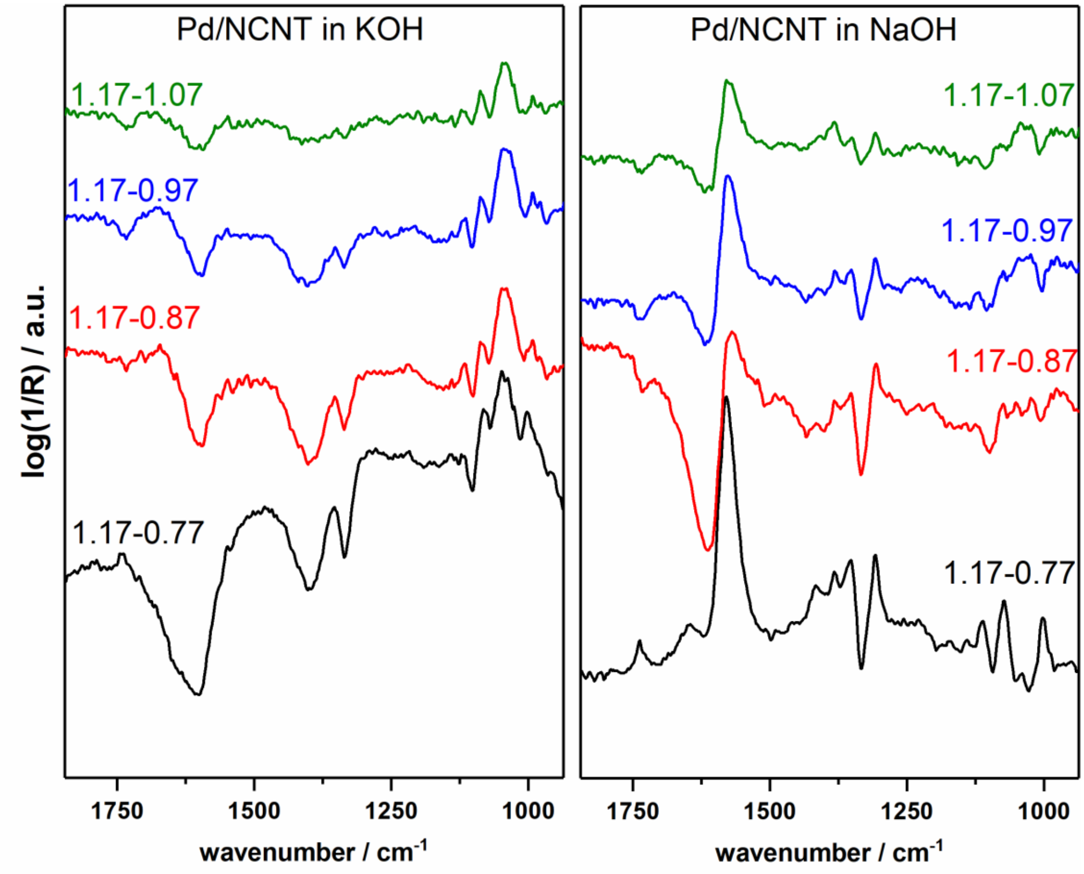

Figure S4: Difference IR spectra for Pd/NCNT of the sample potentials as indicated. The image exhibits difference spectra after the same time of electrolysis of the Pd/NCNT catalyst in NaOH and KOH. It demonstrates the changes between the different potentials. Especially in the 1.17–0.77 V spectrum the differences are rather strong. The spectra in NaOH clearly show that the band at 1335 cm^{-1} is being lost, whereas clear other bands are being formed. The sharp band at 1579 cm^{-1} is assigned to formate and indicates the increasing contribution of this product to the overall spectrum. The corresponding KOH spectra further shows that the 1335 cm^{-1} feature is vanishing with increasing potential. However, the strong negative feature at 1396 cm^{-1} further supports what is mentioned in the main text, i.e., that less carbonate is generated in KOH upon increasing the sample potential.
